# Prediction of findings at screening colonoscopy using a machine learning algorithm based on complete blood counts (ColonFlag)

**DOI:** 10.1371/journal.pone.0207848

**Published:** 2018-11-27

**Authors:** Robert J. Hilsden, Steven J. Heitman, Barak Mizrahi, Steven A. Narod, Ran Goshen

**Affiliations:** 1 Departments of Medicine and Community Health Sciences, Cumming School of Medicine University of Calgary, Calgary, Alberta, Canada; 2 Medial Cancer Research, Kfar Malal, Israel; 3 Familial Breast Cancer Research Unit, Women's College Research Institute, Women's College Hospital, Toronto, ON, Canada; 4 Dalla Lana School of Public Health, University of Toronto, Toronto, ON, Canada; 5 Medial Early Sign, Kfar Malal, Israel; Singapore General Hospital, SINGAPORE

## Abstract

Adenomatous polyps are a common precursor lesion for colorectal cancer. ColonFlag is a machine- learning-based algorithm that uses basic patient information and complete blood cell counts (CBC) to identify individuals at elevated risk of colorectal cancer for intensified screening. The purpose of this study was to determine whether ColonFlag is also able to predict the presence of high risk adenomatous polyps at colonoscopy. This study was conducted at a large colon cancer screening center in Calgary, Alberta. The study population included asymptomatic individuals between the ages of 50 and 75 who underwent a screening colonoscopy between January 2013 and June 2015. All subjects had at least one CBC result within the year prior to colonoscopy. Based on age, sex, red blood cell parameters, inflammatory cells and platelets, the ColonFlag algorithm generated a score from 0 to 100. We compared the ability of the ColonFlag test result to discriminate between individuals who were found to have a high risk polyp and those with a normal colonoscopy. Among the 17,676 individuals who underwent a screening colonoscopy there were 1,014 found to have a high risk precancerous lesion (5.7%) and 60 were found to have colorectal cancer (0.3%). At a specificity of 95%, the odds ratio for a positive ColonFlag was 2.0 for those with an advanced precancerous lesion compared with those with a normal colonoscopy. The odds ratio did not vary according to patient subgroup, colorectal cancer location or stage. ColonFlag is a passive test that can use routine blood test results to help identify individuals at elevated risk for high risk precancerous polyps as well as frank colorectal cancer. These individuals may be targeted in an effort to achieve greater compliance with conventional screening tests.

## Introduction

Population-based screening programs for colorectal cancer have been established in many countries.[1–3] The dual goals of screening are to reduce the incidence or colorectal cancer and subsequent mortality. To achieve these goals, a screening test must detect both high risk precancerous lesions (advanced adenomatous and sessile serrated polyps) and early invasive cancers. The US Multi Society Task Force on Colorectal Cancer Screening ranks colonoscopy and annual fecal immunochemical testing as valid screening options.[1] Due to cost considerations, most population-based screening programs are based on the fecal immunochemical test. Despite the established benefits and the cost-effectiveness of screening for colorectal cancer, the uptake of screening is suboptimal.[4, 5] Colorectal cancer screening requires active participation of the individual by collecting a stool sample and/or undergoing a more invasive test, such as a colonoscopy. Screening could be enhanced through the use of a passive test that uses electronic medical records to identify individuals at elevated risk of harboring an asymptomatic colorectal cancer or a high risk precancerous lesion.

We have described the development and validation of the ColonFlag score (previously known as MeScore), an algorithm that incorporates patient factors (age and gender) with complete blood count information (CBC) and which is used to predict the presence of colorectal cancer at the time of testing.[6–8] ColonFlag was developed using data from healthy Israelis (Maccabi Health Care Services, MHS) and colorectal cancer patients. Training of the model was done using the MHS database and the Israeli National Cancer Registry, which documents invasive colorectal cancer but does not document pre-cancerous lesions. ColonFlag was validated in additional cohorts from Israel (MHS), the UK (Health Information Network database) and US (Kaiser Permanente). In the MHS validation study, the area under the receiver operator curve (AUC) for the detection of colorectal cancer was 0.82 ± 0.01. At a specificity of 90%, 56% of colorectal cancer cases were detected. Similar results were achieved in UK and US study samples.[7, 9] It is expected that the identification and removal of high risk polyps at colonoscopy will enhance efforts to prevent invasive colon cancer. To date, the ability of ColonFlag to predict the presence of high risk precancerous lesions at colonoscopy has not been evaluated.

## Methods

### Study design and setting

This study was conducted at Alberta Health Services’ Forzani & MacPhail Colon Cancer Screening Centre in Calgary, AB, Canada by linking the Centre’s electronic medical records with provincial laboratory data. This study was approved by the Health Research Ethics Board of Alberta (HREBA.CC-16-0162), which waived the requirement for informed consent. The Centre is a publicly-funded endoscopy unit that performs only screening-related colonoscopies. Colonoscopies performed for other indications, including the investigation of signs or symptoms of gastrointestinal disease, such as rectal bleeding or iron deficiency anemia, are done at hospital endoscopy units. The Centre follows published clinical practice guidelines to determine an individual’s eligibility for a first screening or post-polypectomy surveillance colonoscopy. All patients must be free of any medical condition that places them at higher risk for a colonoscopy-related adverse event (predominantly ASA Class I/II). All patients undergo consultation with a trained nurse, and those who do not meet the Centre’s eligibility criteria (for example, those with signs or symptoms of gastrointestinal disease or new anemia) are directed elsewhere. Colonoscopies at the Centre are performed by experienced gastroenterologists and colorectal surgeons who also perform endoscopies at hospital endoscopy units.

### Study population

The study population included individuals between the ages of 50 and 75 who underwent a colonoscopy at the Centre between January 2013 and June 2015. To be included in the study, the patient must have undergone a successful colonoscopy (complete to the cecum unless incomplete due to an obstructing mass) with a bowel preparation rated by the endoscopist as adequate to detect polyps greater than 5 mm in size.

Three subgroups of patients were eligible for the study (1) individuals at average risk for colorectal cancer, (2) individuals with a personal history of polyps and (3) individuals with a family history of polyps or colorectal cancer. Patients were excluded if they had a positive guaiac or immunochemical fecal occult blood test, a prior history of colorectal cancer, a known or suspected genetic predisposition to cancer or no CBC result within the year prior to their colonoscopy.

### Data sources

We obtained data on colonoscopies from the Centre’s endoscopy reporting program endoPRO (Pentax Medical). Data elements included age, sex, date of procedure, indication, depth of endoscope insertion, bowel preparation quality and unique lifetime identifier. Pathology data was obtained from the Centre’s Pathology Database, which includes a structured summary of the pathology report. The summary is completed by a trained nurse who reconciles each polyp reported at colonoscopy with the pathology report. This provides a best possible classification of each resected polyp for those situations where more than one polyp was included in a specimen container. The Centre also regularly links to the Alberta Cancer Registry to identify all colorectal cancer diagnosed on the Centre’s patients and to obtain staging information using the 6^th^ Edition of the American Joint Committee on Cancer Staging Handbook.[10] Prior to August 2013, the Centre’s Pathology Database did not record the presence of dysplasia in sessile serrated polyps.

Complete blood count results were obtained from Alberta Health Services’ Analytics department through a deterministic record linkage using the patients’ unique lifetime identifier. This department receives all laboratory results performed in Alberta. All CBC results from January 1, 2010 to the date of colonoscopy were obtained. Results of CBCs collected during a hospital stay or emergency room visit were excluded. CBC components could include one or more of the following: hemoglobin, hematocrit, mean corpuscular volume, mean corpuscular hemoglobin, mean corpuscular hemoglobin concentration, red blood cell count, red cell distribution width, white blood cell count, platelet count and count and/or percentage of neutrophils, lymphocytes, monocytes, eosinophils and basophils.

### Colonoscopy outcomes

We classified the outcomes of colonoscopies based on the most advanced lesion found into five categories: (Group 1) invasive colorectal cancer, (Group 2) high risk precancerous lesions (advanced adenomatous polyp or sessile serrated polyp with conventional cytological dysplasia, (Group 3) non-advanced adenomatous polyp or non-dysplastic sessile serrated polyp, (Group 4) non-neoplastic findings (i.e. distal hyperplastic polyps, “polyps” classified as normal tissue) and (Group 5) no finding. An advanced adenoma was defined as one greater than 10 mm in size or containing villous elements or high-grade dysplasia.[11]

### ColonFlag scoring

For this study, the ColonFlag algorithm, previously described for the detection of colorectal cancer, was used. ColonFlag incorporates information about age, sex, hemoglobin, red blood cell parameters, white blood cell parameters and platelets using a computer algorithm to generate a raw score that ranges from 0 to 100.[7] For the calculation of the ColonFlag score, the model requires at a minimum the following six CBC components: hemoglobin, red blood cell count, hematocrit, mean corpuscular volume, mean corpuscular hemoglobin and mean corpuscular hemoglobin concentration. Additional CBC components improve the performance of ColonFlag, but are not mandatory. If a CBC does not include one or more of the inflammatory cell parameters or the platelet count, the algorithm imputes a value for these based on the age- and gender-specific means of the population. Values were missing for 0.1% - 1.8% of the inflammatory cell parameters and 0.3% of platelet counts. For this study, all available CBC results completed prior to the date of the colonoscopy were included.

### Statistical analysis

Statistical analysis was performed using R version 3.0.1 (R Development Core Team, Vienna, Austria) and SPSS version 23.0 (IBM, Armonk, New York). The raw ColonFlag scores were transformed into percentiles, based on the distribution of scores in all the patients included in the analysis. A ColonFlag score was considered positive if it was in the top 10^th^ or 5^th^ percentile, depending on the preset specificity of 90% or 95%, respectively. Bootstrap resampling methods were used to estimate the expected mean sensitivity and odds ratio with associated 95% confidence interval of the model performance for the detection of screening-relevant lesions. For 500 bootstrap samples, we calculated the sensitivity and odds ratio. Mean values for each test characteristic and their associated 95% confidence intervals were determined from the bootstrap sample distribution.

The primary goal of the study was to estimate the performance of the ColonFlag test for identifying patients with advanced precancerous colon lesions. We estimated the odds ratio and associated 95% confidence interval for a positive ColonFlag in patients with colorectal cancer (Group 1) for those with an advanced precancerous lesion (Group 2) and for those with non-advanced adenomas (Group 3). All comparisons were done with respect to those with no findings at colonoscopy as a reference (Group 5). We also estimated these odds ratios for subgroups according to anatomic site and cancer stage. Next, we estimated the sensitivity of the ColonFlag in the detection of either colorectal cancer or and advanced precancerous lesions (Groups 1 and 2 combined) compared to those participants with non-advanced polyps, non-neoplastic findings and no findings (Groups 3, 4 and 5 combined).

## Results

### Patient characteristics

There were 27,685 potentially eligible individuals of whom 10,009 were excluded because no CBC result was available for the preceding 12-month period. The characteristics of the 17,676 individuals included in the final study sample are shown in Table 1. The majority of the individuals were at average risk for colorectal cancer; 9.1% of all potentially eligible individuals were considered to be at higher than average risk because of a previous history of polyps and 21.5% were considered to be at higher risk because of a family history of colorectal cancer (Table 1). There were 60 colorectal cancer cases detected and 1014 high risk precancerous lesions detected in the study group. Among the remaining individuals, 26.3% had (one or more) non-advanced adenoma or a non-dysplastic sessile serrated polyp.

**Table 1:** Study sample: Baseline characteristics and colonoscopy outcomes.

		Average Risk	Personal History of Polyps	Family History of Colon Cancer/Polyps	All Patients
	N	12,251	1,617	3,808	17,676
**Gender, n (%)**					
	Male	5,710 (46.6%)	837 (51.8%)	1,458 (38.3%)	8,500 (45.3%)
	Female	6,541 (53.4%)	780 (48.2%)	2,350 (61.7%)	9,671 (54.7%)
**Age Group, n (%)**					
	50–59	7600 (62.0%)	591 (36.5%)	2524 (66.3%)	10,715 (60.6%)
	60–75	4651 (38.0%)	1026 (63.5%)	1284 (33.7%)	6,961 (39.4%)
**Most Recent Laboratory Values**					
	Hemoglobin, mean (SD)	14.52 (1.19)	14.62 (1.19)	14.45 (1.19)	14.51 (1.2)
	WBC, mean (SD)	6.06 (1.73)	6.29 (1.84)	6.13 (1.73)	6.09 (1.7)
	Platelets, mean (SD)	229.6 (55.5)	227.6 (57.5)	234.7 (55.5)	230.5 (55.7)
**Most Advanced Outcome, n (%)**					
	colorectal cancer	30 (0.2%)	10 (0.6%)	20 (0.5%)	60 (0.3%)
	Advanced precancerous	520 (4.2%)	301 (18.6%)	193 (5.1%)	1,014 (5.7%)
	Non-advanced adenoma/SSP	3,036 (24.8%)	569 (35.2%)	1,041 (27.3%)	4,646 (26.3%)
	Non-neoplastic findings	2,177 (17.8%)	315 (19.5%)	760 (20.0%)	3,252 (18.4%)
	No findings	6,488 (53.0%)	422 (26.1%)	1,794 (47.1%)	8,704 (49.2%))

WBC: White Blood Cell Count; colorectal cancer: Colorectal Cancer; SSP: sessile serrated polyp

### ColonFlag test performance

Compared to those with no findings at colonoscopy, the odds ratio for a positive ColonFlag test at a specificity of 95% was 5.1 (95% CI 2.3–8.9) for colorectal cancer, was 2.0 (95% CI 1.6–2.6) for an advanced precancerous lesion, and was 1.7 (95% CI 1.5–2.0) for those with a non-advanced adenoma/serrated polyp. The results at specificities at 90% and at 95% are shown in Table 2.

**Table 2:** Odds ratio of a positive colonflag, by patient subgroup.

Group	Outcome	Number of outcomes	ColonFlag Mean (SD)	Specificity 95%	Specificity 90%
				Odds Ratio	Odds Ratio
**All Patient**					
	colorectal cancer	60	62.7 (19)	5.1 [2.3–8.9]	4.6 (2.5–7.7)
	Advanced adenoma/SSP	1014	56.4 (18.4)	2.0 (1.6–2.5)	2.1 (1.8–2.5)
	Non-Advanced adenoma/SSP	4,646	54.7 (17.7)	1.7 (1.5–2.0)	1.8 (1.6–2.0)
	Non-Neoplastic polyp	3,252	50.7 (17.2)	1.2 (1.0–1.4)	1.2 (1.0–1.3)
	No Findings	8,704	48.5 (16.4)	referent	referent
**Average Risk**					
	colorectal cancer	30	61.9 (17.8)	5.4 (1.2–11.4)	4.6 (1.6–8.5)
	Advanced adenoma/SSP	520	56.5 (17.8)	2.2 (1.6–2.9)	2.0 (1.5–2.5)
	Non-Advanced adenoma/SSP	3,036	54.9 (17.4)	1.8 (1.5–2.1)	1.9 (1.6–2.1)
	Non-neoplastic polyp	2.177	50.9 (16.9)	1.2 (1.0–1.5)	1.2 (1.0–1.4)
	No Findings	6,488	48.4 (16.2)	referent	referent
**Family History**					
	colorectal cancer	20	58.5 (19.0)	5.3 (0.8–12.7)	3.9 (0.9–9.0)
	Advanced adenoma/SSP	193	52.3 (17.9)	2.0 (1.0–3.4)	1.9 (1.2–2.7)
	Non-Advanced adenoma/SSP	1,014	50.5 (16.7)	1.5 (1.0–2.1)	1.5 (1.2–1.8)
	Non-Neoplastic polyp	760	46.9 (15.7)	10.9 (0.6–1.3)	1.0 (0.7–1.3)
	No Findings	1,794	46.0 (16.0)	referent	referent
**Personal History**					
	colorectal cancer	10	73.4 (17.4)	4.1 (0.0–12.9)	4.4 (0.0–13.4)
	Advanced adenoma/SSP	301	59.1 (19.3)	1.0 (0.5–1.6)	0.9 (0.5–1.3)
	Non-Advanced adenoma/SSP	569	61.2 (18.6)	1.4 (0.8–2.0)	1.1 (0.7–1.7)
	Non-Neoplastic Polyp	315	58.4 (19.0)	1.1 (0.6–2.0)	1.1 (0.6–1.6)
	No Findings	422	59.8 (17.8)	referent	referent

colorectal cancer: Colorectal cancer; SSP: sessile serrated polyp

Table 3 shows the sensitivity of ColonFlag for colorectal cancer or an advanced precancerous lesion in the full patient cohort. In this analysis, those with a non-advanced polyp, a non-neoplastic polyp or no findings at colonoscopy are included in the disease-free group. For colorectal cancer, there were no significant differences in sensitivity by anatomic site or cancer stage, although the study had limited power to detect a meaningful difference (Table 4).

**Table 3:** Sensitivity and odds ratio of a positive colonflag for colorectal cancer or an advanced precancerous lesion in all study patients, by patient subgroup.

Group	N	ColonFlag Mean (SD)	CRC/Advanced Precancerous Lesion	Specificity 95%		Specificity 90%	
				Sensitivity	Odds Ratio	Sensitivity	Odds Ratio
**All Patients**	17,676	56.8 (18.5)	1074	8.1 (6.4–9.8)	1.7 (1.3–2.1)	16.8 (14.5–19.0)	1.8 (1.5–2.1)
**Average Risk **	12,251	56.8 (17.9)	550	8.3 (5.7–10.8)	1.7 (1.1–2.3)	16.1 (12.9–19.6)	1.7 (1.3–2.2)
**Family History **	3,808	52.9 (18.1)	213	8.6 (4.7–13.6)	1.8 (0.9–3.0)	16.5 (11.6–22.0)	1.8 (1.2–2.5)
**Personal History **	1,617	59.5 (19.4)	311	4.6 (2.3–7.3)	0.9 (0.4–1.5)	8.7 (5.6–12.5)	0.9 (0.5–1.3)

**Table 4:** Sensitivity and odds ratio of colonflag for different categories of colorectal cancer in all study patients.

Colorectal cancers		N	Specificity 95%		Specificity 90%	
			Sensitivity	Odds Ratio	Sensitivity	Odds Ratio
**Site**						
	Ascending/cecum	13	10.8 (0.0–31.3)	2.6 (0.0–8.6)	36.2 (13.3–66.7)	5.9 (1.4–18.0)
	Other	47	13.2 (4.2–24.3)	3 (0.8–6.1)	25.3 (14.0–37.8)	3.1 (1.5–5.5)
**Stage**						
	Stage I/II	43	10.7 (2.6–20.8)	2.3 (0.5–5.0)	27.2 (15.0–40.5)	3.5 (1.6–6.1)
	Stage III/IV	17	18.3 (0.0–38.9)	4.6 (0.0–12.1)	29.2 (7.1–55.6)	4.1 (0.7–11.3)

### ColonFlag test performance

Compared to those with no findings at colonoscopy, the odds ratio for a positive ColonFlag test at a specificity of 95% was 5.1 (95% CI 2.3–8.9) for colorectal cancer, was 2.0 (95% CI 1.6–2.6) for an advanced precancerous lesion, and was 1.7 (95% CI 1.5–2.0) for those with a non-advanced adenoma/serrated polyp. The results at specificities at 90% and at 95% are shown in Table 2.

Table 3 shows the sensitivity of ColonFlag for colorectal cancer or an advanced precancerous lesion in the full patient cohort. In this analysis, those with a non-advanced polyp, a non-neoplastic polyp or no findings at colonoscopy are included in the disease-free group. For colorectal cancer, there were no significant differences in sensitivity by anatomic site or cancer stage, although the study had limited power to detect a meaningful difference (Table 4).

## Discussion

We have previously demonstrated that ColonFlag is able to enhance detection of invasive colorectal cancers[6, 7, 9]. In this study, we have shown that the ColonFlag score is also able to detect high risk precancerous lesions, such as advanced adenomatous polyps. In general, the performance of ColonFlag was comparable across patient subtypes, anatomic locations and stage. ColonFlag appeared to perform less well in participants with a personal history of polyps. This finding is perhaps surprising given that ColonFlag is likely detecting subtle iron deficiency and alterations in the immune system resulting from the lesion, which would not be expected to be different between those with and without a family history of cancer. Further work on validating the ColonFlag performance in this patient group is warranted.

We focused on the sensitivity of ColonFlag for colorectal cancer and high risk precancerous polyps, rather than the detection of adenomas in general. This is consistent with the goals of screening programs. In this context, a detection of a patient with a non-advanced adenoma only using ColonFlag would be considered a false positive We conducted two sets of analyses: one where only those with no findings were included as controls and the second where those with no neoplasia, non-advanced polyps or no findings at colonoscopy were included as controls. There was only a modest difference in the odds ratio between the two, indicating that in clinical practice, the performance of ColonFlag is not influenced to a great degree by the presence of non-advanced adenomas.

The sensitivity of ColonFlag for colorectal cancer is low compared to other well-established screening tests such as colonoscopy or FIT [1, 12]. ColonFlag should not be viewed as a possible substitute for these tests, rather ColonFlag could provide a useful tool in identifying those unscreened individuals at higher risk for harboring a colorectal cancer or high risk precancerous lesion. People who are told that they are at greater risk for colorectal cancer are more likely to undergo screening.[13] Ideally, ColonFlag could be incorporated into an electronic medical record and performed when a CBC blood test is conducted. If a patient’s ColonFlag score exceeded a preset level, that person would be flagged and prioritized for formal screening [14]. In this way, reductions in colorectal cancer incidence and mortality could be achieved through increasing screening uptake among previously unscreened populations.

ColonFlag does not require any action by either the patient or the health care provider. It can act as a passive means to identify individuals at high risk for colorectal cancer or high risk precancerous lesions. However, ColonFlag is limited by the need for at least one recent CBC result.

This study has several limitations. First, detailed information on a patient’s personal or family history of colorectal cancer or polyps was not available. Therefore, these two groups will include a mix of patients with a history of colorectal cancer or polyps in one or more first degree or more distant relatives. Second, we did not have complete characterization of all sessile serrated polyps. The Centre’s pathology database did not routinely record the presence of conventional dysplasia in these polyps prior to 2013. Therefore, some high-risk polyps in the earlier years will be misclassified as non-high risk. However, the prevalence of conventional dysplasia in sessile serrated polyps is low (<5%).[15] Moreover, ColonFlag was trained and developed using a registry of invasive cancers and in the development stage, high risk precancerous lesions were considered negative findings. In the future, re-training of ColonFlag may increase its overall performance. Finally, within this study, eligibility criteria included the absence of gastrointestinal symptoms or unexplained anemia. Therefore, our study patients, classified as average risk for colorectal cancer, are likely to be at slightly lower risk of colorectal cancer than the (unscreened) general population.

It is possible that the performance of future versions of ColonFlag could be improved using a broader array of standard laboratory tests[8] or by increasing the number of risk factors included in the algorithm. For example, smoking, body mass index and family history of colorectal cancer are commonly recorded in a patient’s electronic medical record. Several risk prediction models for colorectal neoplasia have been developed although none have seen widespread validation or adoption.[16] However, combining ColonFlag with additional clinical risk factors routinely available in an EMR could improve its ability to discriminate high risk from low risk patients.

In summary, the ColonFlag model was able to identify individuals at elevated risk of having colorectal cancer or a high risk precancerous polyp using data solely based on routinely collected complete blood cell counts and patient’s age and sex. These findings support the value of ColonFlag to be embedded into laboratory information systems or electronic health records to identify individuals who warrant targeted efforts to enhance screening compliance.
